# Impact of Urgent Versus Early Endoscopy on Outcomes in Acute Upper Gastrointestinal Bleeding: A Retrospective Study

**DOI:** 10.1155/cjgh/4328051

**Published:** 2025-11-30

**Authors:** Katarzyna Stasik, Katarzyna Ferenc, Krystian Partyka, Wojciech Kilisiński, Rafał Filip

**Affiliations:** ^1^ Gastroenterology Clinic, Center for Comprehensive Treatment of Inflammatory, Bowel Disease Regional Hospital No. 2 in Rzeszow, Rzeszow, 35-301, Poland; ^2^ Department of Internal Medicine, Faculty of Medicine, Medical College of Rzeszow University, University of Rzeszow, Rzeszow, 35-959, Poland, ur.edu.pl

**Keywords:** early endoscopy, mortality, rebleeding, upper gastrointestinal bleeding, urgent endoscopy

## Abstract

**Background and Aims:**

Acute upper gastrointestinal (UGI) bleeding from variceal and nonvariceal sources is a leading cause of morbidity and mortality. Although current international guidelines recommend performing endoscopy within 24 h of presentation, the added value of very early (“urgent,” < 6 h) versus “early” (6–24 h) endoscopy remains unclear. Therefore, this study aimed to analyze the impact of urgent versus early endoscopy on clinical outcomes.

**Methods:**

In this retrospective cohort study, we reviewed the medical records of 599 patients admitted to the emergency or gastroenterology department who underwent UGI endoscopy. Patients were stratified by timing, urgent endoscopy within 6 h of specialist consultation and early endoscopy between 6–24 h postconsultation. The protocol was approved by the University of Rzeszów Ethics Committee and conducted in accordance with the Declaration of Helsinki.

**Results:**

Compared to the early group, patients undergoing urgent endoscopy were younger (*p* = 0.013), predominantly male, and had higher baseline heart rates (*p* = 0.0371). Apart from more frequent hypertension in the early group (*p* = 0.0007), comorbiditiy profiles were otherwise similar. The urgent endoscopy group had longer hospital stays (6.88 vs. 5.95, *p* = 0.774), more frequent rebleeding within 7 days (*p* = 0.0213), and slightly higher 30‐day all‐cause mortality rates. Factors associated with poor prognosis included increases in Rockall’s and GB’s scores (42% and 19% higher risk per point, respectively) and the presence of active bleeding (105% higher odds).

**Conclusions:**

In unadjusted analyses, urgent endoscopy (< 6 h) was associated with worse outcomes, likely reflecting the selection of more severely ill patients. After propensity‐score matching for baseline risk factors, however, the timing of endoscopy (< 6 h vs. 6–24 h) was no longer an independent predictor of outcomes. These findings suggest that clinicians’ decisions and outcome differences are driven by overall severity of presentation and comorbid burden, rather than the effect of ultra‐early endoscopy itself.

## 1. Introduction

Upper gastrointestinal (UGI) bleeding (UGIB) is a frequent cause of emergency department (ED) presentation, with an estimated incidence ranging from 45 to 172 per 100,000 individuals annually [1]. Acute bleeding from the UGI tract originating from both variceal and nonvariceal sources is consistently associated with increased morbidity and mortality, with mortality rates up to 14% [2, 3]. Urgent UGI endoscopy in the ED is essential not only for the diagnosis but also for providing immediate endoscopic hemostatic treatment for actively bleeding lesions, thereby potentially improving patient outcomes [2]. However, the optimal timing for endoscopy in managing acute UGIB remains a controversial issue [4]. Proponents argue that immediate endoscopy expedites diagnosis, enables risk stratification, and introduces preemptive measures; others contend that very early procedures within the first 24‐h postadmission provide minimal benefit regarding mortality, length of stay, or rebleeding [5, 6]. The latter group emphasizes that mortality in UGIB often reflects the burden of comorbid illness rather than acute blood loss alone. A pooled review of five randomized trials and 20 observational cohorts showed no mortality benefit when endoscopy was performed within 24 h of presentation [7]. Moreover, subgroup analysis of cohort data revealed increased death risk for procedures performed within 12 h (OR: 1.66, *p* < 0.001, *I* ^2^ = 0). Reflecting these results, current international guidance emphasizes a 24‐h window for endoscopic evaluation in acute UGIB, rather than an ultra‐early (< 12 h) approach [8]. For nonvariceal hemorrhage, the 2021 ESGE guideline issues a strong recommendation (high‐quality evidence) that, after hemodynamic stabilization, endoscopy should be performed within 24 h, and explicitly advises against routine urgent (< 12 h) procedures, as they do not improve patient‐centered outcomes [8]. For variceal bleeding, the British Society of Gastroenterology similarly advises endoscopy within 24 h of presentation, reserving immediate postresuscitation scope only for those with ongoing instability [9]. Decisions regarding endoscopy timing in UGIB should incorporate patients’ hemodynamic status, comorbidities, and laboratory indices to estimate the risk of diverse outcomes. Although many reports show no mortality difference between urgent and delayed endoscopy, these findings warrant caution, given heterogeneity in study designs and data quality. A broad consensus supports endoscopy within 24 h for most UGIB patients, while acknowledging that some may benefit from alternative timing. Whether any observed harm with ultra‐early endoscopy reflects the procedure itself or simply the higher acuity of patients selected for immediate intervention remains unclear. Therefore, recent research has focused on the precise identification of patients with UGIB at sufficiently low risk for adverse outcomes, in whom endoscopic evaluation can be potentially performed in outpatient settings, as well as defining the optimal endoscopic timing for critically ill patients experiencing severe UGIB [10]. Recognizing which patients with acute UGIB require immediate endoscopy versus those who can safely wait remains essential. Preendoscopic risk‐stratification tools (e.g., Glasgow–Blatchford [GB], AIMS65, and MAP) have been evaluated for their ability to predict active bleeding or high‐risk stigmata at endoscopy, but their performance is inconsistent and does not reliably guide timing [11–13].

This retrospective study therefore examined whether urgent versus early endoscopy influences rebleeding within 7 and 30 days, length of hospital stay, red blood cell (RBC) transfusion volume, and 30‐day mortality among ED patients with UGIB.

## 2. Methods

### 2.1. Study Design and Settings

We conducted a retrospective analysis of medical records for 599 consecutive patients who underwent UGI endoscopy after admission either to the ED at the Department of Gastroenterology with the Center for Comprehensive Treatment of Inflammatory Bowel Disease at Clinical Hospital No. 2 in Rzeszow or the department in question, between 2015 and 2022. Our department provides a 24‐h endoscopy service for approximately 250,000 inhabitants of Rzeszow and the surrounding areas, with gastroenterology consultants and gastrointestinal surgeons on the team. All endoscopic procedures are performed with the support of specialized endoscopic nurses.

### 2.2. Study Population

Patients were eligible if they presented with overt signs of acute UGIB: hematemesis, melena, or both, and had a GB score ≥ 2, indicating a higher risk of rebleeding or death. The GB score was calculated from the lowest systolic blood pressure (SBP), highest heart rate (HR), hemoglobin (Hgb), and serum urea levels on admission, along with the presence of melena or syncope and history of hepatic or cardiac failure. We excluded patients who developed UGIB during an inpatient stay on nongastroenterology wards and those admitted electively for nonbleeding indications (e.g., anemia evaluation or surveillance endoscopy). Based on clinical stability, patients were assigned to one of the two timing cohorts:

- Urgent endoscopy: within 6 h of specialist consultation, reserved for life‐threatening bleeding with rapid deterioration in hematological parameters or failure to respond to blood‐product transfusion and fluid therapy.
- Early endoscopy: 6–24 h after consultation, for patients not requiring immediate intervention or in whom it was deferred until physiological and coagulation parameters were stabilized (for example, in those with chronic circulatory failure, suspected Type 2 myocardial infarction, elevated blood alcohol level delaying anesthesia, or immunohematologic crossmatch issues).

All patients were closely monitored for the next 3 days. Evidence of recurrent bleeding (i.e., fresh hematemesis or hematochezia and hypotensive shock) prompted emergency endoscopy and gastrointestinal surgeon consultation.

### 2.3. Pharmacological Treatment

In accordance with current recommendations, all patients received a high‐dose intravenous proton pump inhibitor therapy on admission (80 mg bolus followed by 8 mg per hour infusion). Patients with known or suspected variceal bleeding (liver failure, stigmata of liver cirrhosis, or prior variceal hemorrhage) received vasoactive agents as well as prophylactic antibiotics.

### 2.4. Endoscopy

Endoscopy was performed according to the current guidelines and recommendations. Definitions for recurrent bleeding were adopted from international guidelines [14]. Rebleeding was defined as an Hgb level reduction of at least 2 g/dL after initial stabilization due to persistent bleeding (bleeding that was not successfully controlled at the initial endoscopy) or recurrent bleeding after endoscopic treatment [14–16]. During endoscopy, gastric and duodenal ulcers with active bleeding or with nonbleeding visible vessels were treated with either hemoclip placement, contact thermocoagulation (including argon plasma coagulation [APC]), and with or without epinephrine injection. Esophageal and gastric variceal bleeding was treated with band ligation or/and injection of a tissue adhesive. Repeat endoscopy with hemostatic treatment was performed for patients with overt recurrent bleeding after the initial endoscopic procedure.

### 2.5. Data Collection

Data including age, sex, clinical features, laboratory findings, comorbidities, anticoagulation therapy, Rockall’s and GB’s scores, presence of active bleeding, endoscopy timing and findings, number of packed RBC (pRBC) units transfused, primary endoscopic intervention, embolization, surgery, and relevant comorbidities such as malignancy, cirrhosis, irritable bowel disease, ulcer history, ischemic or hemorrhagic stroke, excessive intake of alcohol, and other were retrieved from medical records. Bleeding activity was classified with the Forrest classification [17]. Rockall’s and GB’s scores were recorded for all patients. The study was approved by the Institutional Ethics Committee of the University of Rzeszow (no. 2023/002 of January 4, 2023) and conducted in accordance with the Declaration of Helsinki and good clinical practice.

### 2.6. Statistical Analysis

Continuous variables are presented as means ± SD, and categoric variables as counts and percentages. Normality of variable distribution was assessed with the Student’s *t*‐test. The chi‐square test evaluated associations between categorical variables, with Fisher’s exact test used for small or unbalanced samples. The Mann–Whitney *U* test was used to compare non‐normal ordinal or continuous variables. Statistical significance was defined as *p* < 0.05 for all analyses.

## 3. Results

Out of 599 patients with overt signs of acute UGIB, 102 underwent urgent endoscopy (< 6 h), and the remaining 497 individuals underwent early endoscopy (6–24 h from admission). Patients in the urgent endoscopy group were significantly younger and more often men. Baseline HR was higher in the urgent group (89.32 ± 15.91 in the urgent group vs. 85.33 ± 16.09 in the early endoscopy group, *p* = 0.0371), which may be associated with physiological compensation for volume loss by increasing HR and contractility. Indeed, patients in the urgent endoscopy group had lower Hgb at admission (8.73 ± 2.98 vs. 8.82 ± 2.69), but the number of pRBC units transfused was similar (2.93 ± 2.78 vs. 2.92 ± 2.65), with no significant between‐group differences. Both groups did not differ significantly in terms of analyzed comorbidities, except for hypertension, which was more frequent in the early endoscopy group. Active bleeding was reported more often in the urgent endoscopy group (49.02% vs. 33.20%,*p* = 0.0035). The comparison of both groups did not reveal significant differences in the occurrence of risk factors, including the use of nonsteroidal anti‐inflammatory drugs or anticoagulant medicines (except for new anticoagulant drugs). However, patients in the urgent endoscopy group were much more frequently admitted due to drinking excessive amounts of alcohol.

Endoscopic findings were broadly similar between groups, with the exception of Mallory–Weiss syndrome, which was more common in the urgent group. Urgent endoscopy is the optimal way to control and manage bleeding in the course of this disease. In nearly 40% of urgent group patients and about 45% of early‐group patients, the cause of bleeding was not identified during endoscopy, and thus intervention was not necessary in a large group of patients. Potential explanations include resolved hemorrhage, sources distal to standard upper‐endoscope reach, and bleeding from tumors localized within UGI, bile ducts, or from the ampulla of Vater or subtle mucosal lesions. Among identified causes, gastric ulcers were the most frequent in both groups. Clips, epinephrine, APC, or other thermal methods were the most common interventions in both groups. Sengstaken–Blakemore tamponade, considered a lifesaving bridge therapy, was performed more frequently in the urgent endoscopy group, which is not surprising.

The characteristics of both groups are presented in Tables 1 and 2.

**Table 1:** Baseline characteristics of both groups.

Parameter	Urgent endoscopy (*N* = 102)	Early endoscopy (*N* = 497)^∗^	*p*
Age (years) [mean ± SD]	63.89 ± 16.86	68.02 ± 16.64	**0.013** ^**U**^
Sex (male) [*N* (%)]	78 (76.47%)	336 (67.61%)	0.079^C^
Systolic blood pressure (mmHg) [mean ± SD]	119.22 ± 21.73	121.58 ± 22.60	0.226^U^
Diastolic pressure (mmHg) [mean ± SD]	69.93 ± 14.70	71.39 ± 13.26	0.333^U^
Heart rate (bpm) [mean ± SD]	89.32 ± 15.91	85.33 ± 16.09	**0.0371** ^**U**^
Rockall’s score [mean ± SD]	4.14 ± 2.10	4.31 ± 2.08	0.501^U^
Glasgow–Blatchford’s score [mean ± SD]	10.30 ± 3.43	10.03 ± 3.10	0.501^U^
Glasgow–Blatchford’s score 2 pts. [*N* (%)]	97 (95.10%)	461 (92.76%)	0.225^C^
Active bleeding [*N* (%)]	50 (49.02%)	165 (33.20%)	**0.0035** ^**C**^
Comorbidities [*N* (%)]			
Hypertension [*N* (%)]	23 (22.54%)	203 (40.85%)	**0.0007** ^**C**^
Ischemic heart disease [*N* (%)]	14 (13.73%)	92 (18.51%)	0.308^F^
Cirrhosis [*N* (%)]	10 (9.80%)	24 (4.83%)	0.083^F^
Renal failure [*N* (%)]	8 (7.84%)	47 (9.46%)	0.736^F^
Ischemic stroke [*N* (%)]	6 (5.88%)	25 (5.03%)	0.083^F^
Tumor [*N* (%)]	2 (1.96%)	10 (2.01%)	0.999^F^
Chronic obstructive pulmonary disease [*N* (%)]	3 (2.94%)	14 (2.82%)	0.999^F^
Irritable bowel syndrome [*N* (%)]	0 (0%)	2 (0.40%)	0.999^F^
History of stomach ulcers [*N* (%)]	6 (5.88%)	25 (5.03%)	0.806^F^
History of duodenal ulcer [*N* (%)]	7 (6.86%)	19 (3.82%)	0.183^F^
History of stomach and duodenal ulcers [*N* (%)]	1 (0.98%)	9 (1.81%)	0.999^F^
Risk factors [*N* (%)]			
NSAIDs [*N* (%)]	4 (3.92%)	35 (7.04%)	0.376^F^
Aspirin [*N* (%)]	2 (1.96%)	18 (3.62%)	0.552^F^
Clopidogrel [*N* (%)]	0 (0%)	1 (0.20%)	0.999^F^
Acenocoumarol/warfarin [*N* (%)]	3 (2.94%)	25 (5.03%)	0.450^F^
New anticoagulant drugs [*N* (%)]	0 (0%)	20 (4.02%)	**0.034** ^**F**^
Hemoglobin on admission (g/dL) [mean ± SD]	8.73 ± 2.98	8.82 ± 2.69	0.997^U^
Hemoglobin at discharge (g/dL) [mean ± SD]	10.20 ± 1.37	10.14 ± 1.23	0.709^U^
INR at admission [mean ± SD]	1.39 ± 1.00	1.45 ± 1.15	0.599^U^
Excessive intake of alcohol [*N* (%)]	26 (25.49%)	64 (12.87%)	**0.0021** ^**F**^

*Note:* The bold values indicate statistical significance values.

Abbreviations: INR, international normalized ratio; NSAIDs, nonsteroidal anti‐inflammatory drugs; SD, standard deviation.

^∗^This category includes causes of bleeding that were not detected during endoscopy, e.g., bleeding from the small intestine, bleeding tumors within the upper gastrointestinal tract, and bleeding from the bile ducts or from the ampulla of Vater.

^C^Chi^2^ test.

^F^Fisher’s test.

^U^Mann–Whitney test.

**Table 2:** Endoscopic examination results and study endpoints.

Endoscopic examination finding	Urgent endoscopy (*N* = 102)	Early endoscopy (*N* = 497)	*p*
No pathological findings identified [*N* (%)]	5 (4.90%)	14 (2.82%)	0.345^C^
Stomach ulcer [*N* (%)]	22 (21.57%)	196 (39,44%)	1.000^C^
Duodenal ulcer [*N* (%)]	31 (30.39)	134 (26.96)	0.468^C^
Ulcer in the anastomosis [*N* (%)]	0 (0%)	2 (0.40%)	0.4294
Esophageal ulcer/erosion [*N* (%)]	2 (1.96%)	28 (5.63%)	0.1399^C^
Dieulafoy’s lesion [*N* (%)]	2 (1.96%)	28 (5.63%)	0.1399
Esophageal varices [*N* (%)]	1 (0.98%)	7 (1.41%)	1.000^C^
Varicose veins of the fundus of the stomach [*N* (%)]	5 (4.90%)	22 (4.43%)	0.7947^F^
Hemorrhagic inflammation^&^ [*N* (%)]	0 (0%)	1 (0.20%)	0.3118^F^
Mallory–Weiss syndrome [*N* (%)]	14 (13.72%)	28 (5.63%)	**0.0088** ^**F**^
Other^∗^ [*N* (%)]	38 (37.25%)	224 (45.07%)	0.1557^C^
Mortality within 30 days [*N* (%)]	7 (6.86%)	22 (4.43%)	0.3102^F^
Rebleeding < 7 days [*N* (%)]	7 (6.86%)	11 (2.21%)	**0.0213** ^**F**^
Rebleeding < 30 days [*N* (%)]	1 (0.98%)	8 (1.61%)	1.000^F^
Endoscopic treatment of bleeding [*N* (%)]			
No intervention^#^	50 (49.02%)	306 (61.57%)	**0.0187** ^**C**^
Adrenalin [*N* (%)]	16 (15.69%)	72 (14.49%)	0.0971^C^
APC or other thermal methods [*N* (%)]	12 (11.76%)	47 (9.46%)	0.5077^C^
Clips [*N* (%)]	32 (31.37%)	123 (24.75%)	0.1641^C^
Banding [*N* (%)]	6 (5.88%)	15 (3.02%)	0.148^F^
Surgical treatment			
Surgical lining of the ulcer [*N* (%)]	3 (2.94%)	5 (1.01%)	0.1404^F^
Angiography/embolization [*N* (%)]	1 (0.98%)	0 (0%)	0.3118^F^
Sengstaken–Blakemore probe [*N* (%)]	4 (3.92%)	2 (0.40%)	**0.0091** ^**F**^
Hospitalization time (days) [mean ± SD]	6.88 ± 5.39	5.95 ± 3.53	0.774^U^
RBC transfusion (units) [mean ± SD]	2.93 ± 2.78	2.92 ± 2.65	0.844^U^
Number of plasma units transfused (mean ± SD)	1.03 ± 1.87	0.64 ± 1.16	0.127^U^

*Note:* The bold values indicate statistical significance values.

Abbreviations: APC, argon plasma coagulation; SD, standard deviation.

^∗^Gastric tumors and vascular malformations, e.g., angiectasis, gastritis, duodenitis, and esophagitis.

^#^Patients who no longer required intervention upon admission (with Forrest score below IIb and IIc).

^&^Refers to a hemorrhagic inflammatory state of the mucosa, in which the lining is friable and bleeds easily, often with visible bleeding erosions or ulcerations.

^C^Chi^2^ test.

^F^Fisher’s test.

^U^Mann–Whitney test.

### 3.1. Endpoints

All‐cause 30‐day mortality was slightly higher in the urgent endoscopy group; however, the difference between the two groups was not statistically significant: 7 deaths (6.86%) in the urgent endoscopy group vs. 22 deaths (4.43%) in the early endoscopy group (*p* = 0.310). Rebleeding within < 7 days occurred more often after urgent endoscopy compared to the early procedure (6.86% vs. 2.21%,*p* = 0.021); however, the rebleeding rate within 30 days did not differ significantly between groups. The mean length of hospitalization was similar (6.88 vs. 5.95 days;*p* = 0.774).

The summary of study endpoints is presented in Table 2.

### 3.2. Variceal vs. Nonvariceal Bleeding

We also compared baseline characteristics, management, and outcomes of patients with variceal bleeding compared to those with nonvariceal bleeding. The results are presented in Table 3. Patients with variceal bleeding were younger and far more likely to have cirrhosis and a history of alcohol misuse, whereas hypertension predominated in the nonvariceal bleeding group. Despite similar admission HRs, GB scores, and initial Hgb levels, patients with varicose vein bleeding had higher Rockall scores and were more frequently treated with band ligation or Sengstaken–Blakemore tamponade, while nonvariceal bleeding cases more commonly received epinephrine injection or thermal coagulation.

**Table 3:** Baseline characteristics, management, and outcomes of patients with variceal versus nonvariceal upper gastrointestinal bleeding.

Parameter	Varicose vein bleeding (*N* = 59)	Nonvaricose vein bleeding (*N* = 540)	*p*
Age [mean ± SD]	57.53 ± 12.90	68.39 ± 16.77	**< 0.0001**
Urgent endoscopy [*N* (%)]	14 (23.73%)	88 (16.30%)	0.148
Sex (male) [*N* (%)]	45 (76.27%)	369 (68.33%)	0.237
Heart rate (bpm) [mean ± SD]	85.22 ± 16.59	86.03 ± 16.11	0.583
Rockall’s score [mean ± SD]	5.78 ± 1.83	4.12 ± 2.05	**< 0.0001**
Glasgow–Blatchford’s score [mean ± SD]	9.91 ± 2.70	9.99 ± 3.61	0.604
Active bleeding [*N* (%)]	26 (44.07%)	189 (35.00%)	0.1981
Comorbidities [*N* (%)]			
Hypertension [*N* (%)]	12 (20.34%)	214 (39.63%)	**0.0044**
Cirrhosis [*N* (%)	28 (49.46%)	6 (1.02%)	**< 0.00001**
Renal failure [*N* (%)]	3 (5.08%)	52 (9.63%)	0.3436
Ischemic stroke [*N* (%)]	0 (0%)	31 (5.74%)	0.2372
Tumor [*N* (%)]	2 (3.39%)	10 (1.85%)	0.2962
Chronic obstructive pulmonary disease [*N* (%)]	0 (0%)	17 (3.15%)	1.000
Risk factors			
NSAIDs [*N* (%)]	2 (3.39%)	38 (7.04%)	0.162
Aspirin [*N* (%)]	0 (0%)	20 (3.70%)	0.711
Clopidogrel [*N* (%)]	0 (0%)	1 (0.20%)	0.1874
Acenocoumarol/warfarin [*N* (%)]	1 (1.69%)	27 (5.00%)	0.5092
New anticoagulant drugs [*N* (%)]	1 (1.69%)	19 (3.52%)	0.710
Hemoglobin on admission (g/dL) [mean ± SD]	8.57 ± 2.31	8.39 ± 2.74	0.708
Hemoglobin at discharge (g/dL) [mean ± SD]	9.54 ± 1.28	10.22 ± 1.23	0.0002
INR at admission [mean ± SD]	8.57 ± 2.31	8.39 ± 2.74	0.617
Excessive intake of alcohol [*N* (%)]	30 (50.85%)	60 (11.11%)	**< 0.00001**
Mortality within 30 days [*N* (%)]	1 (1.69%)	28 (5.19%)	0.345^F^
Rebleeding < 7 days [*N* (%)]	2 (3.39%)	16 (2.96%)	0.6906^F^
Rebleeding < 30 days [*N* (%)]	1 (1.69%)	8 (1.48%)	0.6034^F^
Endoscopic treatment of bleeding			
No intervention^#^ [*N* (%)]	29 (49.25%)	327 (60.56%)	0.095^C^
Adrenalin [*N* (%)]	1 (1.69%)	87 (16.11%)	**0.0014** ^**C**^
APC or other thermal methods [*N* (%)]	1 (1.69%)	58 (10.74%)	**0.0208** ^**C**^
Clips [*N* (%)]	12 (20.34%)	143 (26.48%)	0.3503^C^
Banding [*N* (%)]	17 (28.81%)	4 (0.74%)	**< 0.00001** ^**F**^
Surgical treatment			
Surgical lining of the ulcer [*N* (%)]	0 (0%)	8 (1.48%)	0.6093^F^
Angiography/embolization [*N* (%)]	0 (0%)	1 (0.20%)	0.1874
Sengstaken–Blakemore probe [*N* (%)]	4 (6.78%)	2 (0.37%)	**0.0011**
Hospitalization time (days) [mean ± SD]	6.88 ± 5.39	5.76 ± 2.71	0.941
RBC transfusion (units) (mean ± SD)	2.39 ± 2.67	3.11 ± 2.32	**0.031**
Number of plasma units transfused (mean ± SD)	1.03 ± 2.17	0.66 ± 1.19	0.494

*Note:* The bold values indicate statistical significance values.

^#^Patients who no longer required intervention upon admission (with Forrest score below IIb and IIc)

^C^Chi_2_ test.

^F^Fisher’s test.

### 3.3. Predictors of Prognosis

We also performed univariate and multivariate analyses to assess whether any of the analyzed parameters were associated with poor prognosis, defined as rebleeding within 30 days or mortality. Univariate analysis showed that a 10‐year increase in patients’ age was associated with an 18% higher risk of composite poor outcome (rebleeding within 30 days or death). Moreover, each 1‐point increase in Rockall’s score and GB’s score was associated with 67% and 25% higher risk of poor prognosis, respectively. Also, the presence of active bleeding tripled the risk, while a cancer diagnosis within 5 years from admission to the hospital due to bleeding increased this risk 3.62‐fold. Poor prognosis was also associated with renal failure (increased by 4.31 times) and a 10‐beat/min increase in heart ratio, which translated into 9% higher risk. The results of the univariate analysis are summarized in Table 4. Variables significant in the univariate analysis were entered into the multivariate analysis. This analysis confirmed that Rockall’s scores, GB’s scores, as well as the presence of active bleeding were independent predictors of poorer patients’ prognosis. The results are summarized in Table 5.

**Table 4:** The impact of selected parameters on patient’s prognosis (univariate analysis).

Parameter	OR	95% CI	*p*
Age (per 10 years)	1.18	0.98–1.42	0.073
Sex (male)	1.08	0.52–2.23	0.840
Rockall’s score (points)	1.67	1.42–1.97	**< 0.0001**
Glasgow–Blatchford score (points)	1.25	1.14–1.38	**< 0.0001**
Active bleeding	3.12	1.75–5.54	**< 0.0001**
Tumor within the last 5 years	3.62	0.95–13.75	0.059
Renal failure	4.31	2.17–8.57	**< 0.0001**
History of stomach ulcers	1.09	0.32–3.70	0.892
NSAIDs	0.83	0.25–2.80	0.768
Aspirin	1.13	0.25–5.00	0.874
Acenocoumarol/warfarin	0.77	0.18–3.33	0.726
New anticoagulant drugs	1.83	0.52–6.46	0.347
Hgb at admission	0.94	0.85–1.05	0.268
Heart rate (each 10 beats per‐minute increase)	1.09	0.92–1.30	0.321
Excessive intake of alcohol	0.80	0.35–1.82	0.590

*Note:* Hgb, hemoglobin. The bold values indicate statistical significance values.

Abbreviations: CI, confidence interval; NSAIDs, nonsteroidal anti‐inflammatory drugs; OR, odds ratio.

**Table 5:** The impact of selected parameters on patients’ prognosis (multivariate analysis).

Parameter	OR	95% CI	*p*
Sex (male)	1.12	0.54–2.32	0.76
Age (10 years)	0.89	0.67–1.19	0.430
Rockall’s score (points)	1.42	1.15–1.75	**0.001**
Glasgow–Blatchford’s score (points)	1.19	1.03–1.37	**0.018**
Active bleeding	2.05	1.04–4.04	**0.039**
Tumor within the last 5 years	1.37	0.60–3.13	0.45
Renal failure	2.49	1.03–6.02	**0.043**
Heart rate (each 10 beats per‐minute increase)	0.97	0.82–1.15	0.71

*Note:* Multivariate analysis including all factors that were significant or on the edge of significance in the univariable model. The bold values indicate statistical significance values.

Abbreviations: CI, confidence interval; OR, odds ratio.

### 3.4. Predictors of Urgent Endoscopy

We also performed univariate and multivariate analyses to identify predictors of the urgent endoscopy. In univariable analyses, each 10‐year increase in age was associated with a 13% lower likelihood of urgent endoscopy and a 34% lower likelihood of ischemic heart disease. Conversely, male sex increased the chances for urgent endoscopy by 1.64 times, active bleeding by 1.92 times, excessive intake of alcohol by 2.3 times, and each 10‐beat/min increase in HR by 16%. These results are summarized in Table 6. In the multivariable model, only active bleeding, excessive alcohol intake, and HR remained statistically significant (Table 7).

**Table 6:** The impact of selected parameters on the decision to choose urgent endoscopy (univariate analysis).

Parameter	OR	95% CI	*p*
Age (10 years)	0.87	0.77–0.98	**0.025**
Sex (male)	1.64	1.02–2.64	**0.039**
Rockall’s score (points)	0.97	0.87–1.07	0.518
Glasgow–Blatchford score (points)	1.03	0.97–1.10	0.321
Active bleeding	1.92	1.25–2.96	**0.003**
Renal failure	0.82	0.37–1.78	0.612
Tumor within 5 years	1.09	0.23–5.11	0.916
Ischemic heart disease	0.56	0.36–0.87	**0.011**
History of stomach ulcers	1.42	0.76–2.67	0.271
NSAIDs	0.54	0.19–1.56	0.256
Aspirin	0.54	0.12–2.34	0.408
Acenocoumarol/warfarin	0.61	0.18–2.06	0.427
Hgb at admission	0.98	0.911–1.06	0.607
INR	0.96	0.79–1.15	0.209
Heart rate (each 10 beats per‐minute increase)	1.16	1.02–1.32	**0.027**
SBP (per 10 mmHg)	0.95	0.87–1.05	0.344
DBP (per 10 mmHg)	0.91	0.78–1.07	0.252
Excessive intake of alcohol	2.29	1.38–3.81	**0.001**

*Note:* Hgb, hemoglobin. The bold values indicate statistical significance values.

Abbreviations: CI, confidence interval; INR, international normalized ratio; NSAIDs, nonsteroidal anti‐inflammatory drugs; OR, odds ratio.

**Table 7:** The impact of selected parameters on the decision to choose urgent endoscopy (multivariate analysis).

Parameter	OR	95% CI	*p*
Age (10 years)	1.05	0.89–1.23	0.573
Sex (male)	0.99	0.86–1.16	0.973
Heart rate (each 10 beats per‐minute increase)	1.16	1.00–1.35	**0.046**
Active bleeding	2.57	1.52–4.34	**0.0004**
Ischemic heart disease	0.73	0.44–1.21	0.224
Alcohol intake	2.12	1.16–3.88	**0.015**

*Note:* The bold values indicate statistical significance values.

Abbreviations: CI, confidence interval; OR, odds ratio.

### 3.5. Outcomes by Endoscopic Therapy

The analysis of the association between endoscopic therapy mode and the specified outcomes failed to bring any significant results. Within 30 days, death occurred in 4 patients (4.0%) following ulcer therapy (including epinephrine, thermal methods, or clips) and in 2 patients (6.2%) in the variceal therapy group (*p* = 0.63). Rebleeding within 30 days occurred in one patient (1.0%) after ulcer therapy and in none of the patients who underwent variceal therapy (*p* = 1.00).

### 3.6. Propensity‐Score Matching

Once we balance on the full set of baseline covariates via propensity‐score matching, none of the candidate variables (active bleeding, HR, alcohol misuse, risk scores, comorbidities, medications, etc.) remained significant predictors of an urgent endoscopy decision (Supporting Tables 1 and 2; Supporting Figure 1). Variables that appeared influential before matching no longer stood out after matching like‐with‐like patients with very similar overall risk profiles, indicating that overall risk constellation, rather than any single variable in isolation, guided timing decisions.

## 4. Discussion

Clinical guidelines frequently advocate for endoscopy within 24 h for patients with suspected nonvariceal UGIB presenting to the ED [18]. However, the breadth of this 24‐h window fuels debates on the utility of urgent endoscopy, specifically performed within six hours. Our study compared urgent versus early endoscopy with respect to mortality, rebleeding risk, and the length of hospitalization.

In our study, most patients presented with hematemesis, melena, and features of hemorrhagic shock. Similarly, Olivarec‐Bonill et al. [19] observed that clinical manifestations of UGIB predominantly encompassed hematemesis (92.2%) and melena (46.5%). Although the bleeding source was not identified during endoscopy in a large portion of our cohort, stomach ulcers were the most frequently detected pathology. According to the literature, peptic ulcers are responsible for 40% to 50% of UGIB cases, with duodenal ulcers contributing to 30% of them [20]. Peptic ulcers are commonly associated with NSAID use, *Helicobacter pylori* infection, and stress‐related mucosal disease [21, 22]. Other causes of UGIB include erosive esophagitis (11%), duodenitis (10%), varices (5%–30%), Mallory–Weiss tears (5% to 15%), and vascular malformations (5%) [20]. In our study, the occurrence of risk factors, including the exposure to NSAIDs, antiplatelet or anticoagulant therapy, and underlying liver disease, was low in both groups. By contrast, in an international cohort study of 619 patients requiring endoscopic intervention, 44% of patients with bleeding were on either antiplatelet or anticoagulant therapies upon admission, with 25% being on both [23]. Nearly one‐fourth of our urgent endoscopy cohort reported excessive alcohol consumption. Kärkkäinen et al. [24] reported that alcohol misuse was associated with a twofold increase in the risk of rebleeding, after adjusting for comorbidities. They also observed a trend toward higher overall mortality among alcohol abusers compared to nonabusers.

In our study, hospitalization duration and RBC transfusion requirement were higher in the urgent endoscopy group compared to the early endoscopy group; however, these differences did not reach statistical significance. Similarly, Ramos et al. [18] demonstrated significant differences between the two groups in both transfusion rates (57.5% vs. 68.4%; *p* < 0.001) and the volume of transfused RBC concentrates (2.85 ± 4.01 vs. 3.51 ± 4.09, *p* = 0.008). Also, Cho et al. [3] found that parameters such as the amount of transfused pRBCs, the requirement for endoscopic intervention, and radiological embolization were notably higher in urgent compared to elective (6–48 h) endoscopy groups. The marked differences observed between patients with variceal and nonvariceal bleeding in our study highlight their differing pathophysiology and management needs. Patients with variceal hemorrhage were much younger but exhibited a markedly higher burden of cirrhosis and alcohol misuse, likely explaining their elevated Rockall scores despite comparable GB scores and admission Hgb levels. Their preference for band ligation reflects established recommendations, favoring endoscopic variceal ligation, whereas nonvariceal bleeders more often underwent injection or thermal therapy [25, 26].

Our univariate analysis identified male sex, active bleeding, excessive intake of alcohol, and elevated HR as predictors of urgent endoscopy. Conversely, older age and ischemic heart disease were associated with lower odds of urgent endoscopy. However, in the multivariable analysis, only active bleeding, excessive intake of alcohol, and HR remained statistically significant, consistent with previous findings [24, 27, 28]. Other studies have shown that features warranting urgent endoscopy include hemodynamic shock with hypotension and tachycardia, GB score of 7–12, persistent in‐hospital hematemesis, bloody nasogastric aspirate, as well as comorbid conditions (e.g., cirrhosis) [3, 29]. Cho et al. [3] suggested that urgent endoscopy should be considered in high‐risk patients with nonvariceal UGIB and with a GB score of > 7. However, in our study, neither GB’s nor Rockall’s scores correlated significantly with the timing of the endoscopic procedure. The GB’s score, preendoscopy Rockall’s score, and AIMS65 are common tools used to stratify UGIB patients into low‐risk and high‐risk categories [8, 30]. GB’s score is more effective than the other two in predicting the need for endoscopic procedures, blood transfusions, radiological and surgical interventions, or mortality [31]. Many healthcare facilities use GB’s score upon UGIB presentation, and patients with a GB score of ≤ 1 often receive early outpatient diagnostic endoscopy instead of hospitalization [10]. However, a study of 3012 UGIB patients revealed that GB’s, AIMS65, and preendoscopy Rockall’s scores have limited accuracy in predicting the need for endoscopic therapy and mortality (AUROCs < 0.80) [31]. Although a GB score of 7 had the best predictive value for endoscopic intervention, its AUROC of 0.75 limits its clinical use. To improve the identification of patients with acute UGIB who need early urgent UGI endoscopy, Adamopoulos et al. [2] developed a new scoring system. Their approach is based on four independent predictors, achieving a sensitivity of 96%, a specificity of 98%, a positive predictive value of 96%, and a negative predictive value of 98% [2]. In general, the European Society of Gastrointestinal Endoscopy and the American Society for Gastrointestinal Endoscopy guidelines recommend early endoscopy within 24 h for most hospitalized UGIB patients, while urgent endoscopy (within 12 h) should be reserved for those with high‐risk clinical features [3, 24]. These guidelines are in line with the American College of Gastroenterology’s recommendations, which emphasize the importance of resuscitation to stabilize hemodynamic parameters and management of other medical conditions before endoscopy [27].

We also evaluated predictors of rebleeding and mortality. In our cohort, rebleeding within 7 days was more common in the urgent endoscopy group with a similar trend at 30 days. Previous studies have reported inconsistent results. Cho et al. [3] found recurrent bleeding to be strongly associated with high‐risk endoscopic lesions, specifically Forrest I and II ulcers, as well as coagulopathy. The authors observed that active bleeding, when untreated endoscopically, resulted in high rebleeding (55%) and mortality (11%) rates [3]. Interestingly, the type of endoscopic therapy did not affect the risk of rebleeding. In turn, Guan et al. [32] reported that performing endoscopy within 12 h did not improve outcomes for patients with nonvariceal UGIB and may even increase the rebleeding rate in low‐risk patients. This aligns with the findings of a systematic review with meta‐analysis, which found no significant difference in overall or subgroup rebleeding rates between early (< 12 h) and delayed endoscopy groups, nor in in‐hospital or 6‐week rebleeding outcomes [33]. Their analysis further indicated that early endoscopy did not reduce the need for additional hemostasis, transfusion requirements, or length of stay. Such findings align with ours, suggesting that urgent interventions may not guarantee improved hemostatic or rebleeding outcomes in unselected patients.

We observed higher all‐cause 7‐ and 30‐day mortality rates in patients in the urgent endoscopy group. This may reflect that mortality in UGIB is largely driven by comorbidities rather than the technical success of hemostasis [34]. Moreover, endoscopy carries certain risks, including cardiopulmonary complications, infections, thromboembolic events, bleeding, and mechanical issues such as perforation, penetration, and impaction, as well as adverse drug reactions from premedication [35, 36]. The evidence regarding the timing of endoscopy remains conflicting; while some studies show no significant difference in mortality between urgent and elective endoscopy, others report reduced mortality with urgent endoscopy in high‐risk patients with acute nonvariceal UGIB [3, 29, 36, 37]. Similarly to our results, Lau et al. [15] demonstrated that endoscopy performed within 6 h after gastroenterological consultation did not reduce mortality compared with procedures conducted at 6–24 h in stable patients hospitalized with acute UGIB who were at high risk of further bleeding and death. In turn, a retrospective analysis of 240 adults with nonvariceal UGIB and a high‐risk GB score (≥ 12) admitted to the ED and undergoing endoscopic examination (63%, urgent endoscopy within < 12 h; 37%, early endoscopy within 12–24 h) found no significant differences in the composite outcome, including 30‐day mortality, rebleeding, endoscopic reintervention, surgical interventions, or angiographic embolization, between the two groups [38]. Bai et al. [33] similarly reported that early endoscopy (< 12 h) was associated with significantly lower overall mortality in pooled analysis (odds ratio [OR] = 0.56, 95% confidence interval [CI]: 0.33–0.95); however, this mortality benefit was no longer significant after subgroup stratification. This partially supports our findings, in which earlier intervention conferred no statistically significant survival advantage, suggesting that factors beyond procedural timing, such as hemodynamic stabilization, comorbidities, and bleeding etiology, may be more decisive determinants of outcome. In contrast, Peng et al. [39] focused on cirrhotic patients with acute variceal bleeding and demonstrated that endoscopy performed within 48 h was associated with a higher rate of 5‐day failure to control bleeding (9.7% vs. 2.4%), while in‐hospital mortality remained similar across timing thresholds (< 12, < 24, and < 48 h). These results echo our observation that earlier intervention does not necessarily translate into superior outcomes, particularly in patients with variceal or advanced liver disease, where adequate resuscitation and stabilization before the procedure may be more crucial than the exact timing of endoscopy.

Lim et al. [29] examined whether high‐risk patients could benefit from endoscopy performed earlier than the recommended 24 h target. Among patients with a GB score of ≥ 12 who died, the time from presentation to endoscopy was significantly longer than in survivors. Multivariate analysis revealed that high‐risk patients who underwent endoscopy more than 13 h after presentation had significantly higher all‐cause in‐hospital mortality compared to those who had the procedure within 13 h (44% vs. 0%, *p* < 0.001) [29]. Consistent with these findings, a prospective observational study comparing outcomes of urgent (< 6 h) and early endoscopy (6−24 h) found that urgent intervention did not reduce 30‐day mortality among patients with acute UGIB or those at high GB scores (≥ 12). However, in patients exhibiting high‐risk endoscopic lesions (Forrest I‐IIB), urgent endoscopy emerged as a vital predictor of reduced mortality [18]. In this study, no statistically meaningful disparities were observed in the rebleeding rate, or the need for further endoscopic interventions [18]. In contrast, a prospective cohort study including 961 consecutive patients with a GB score of > 7 who underwent endoscopy for UGIB demonstrated marked differences in mortality rates between the urgent (< 6 h) and elective (6–48 h) groups (1.6% vs. 3.8%, respectively), with urgent endoscopy strongly associated with lower mortality rate among high‐risk patients [3]. The authors further observed that mortality among patients with rebleeding occurred exclusively in those subjected to endoscopic retreatment or those who received no further treatment, while patients managed with embolization or surgical interventions experienced no fatalities [3]. Patients who received no further treatment displayed an exceptionally high mortality rate of 36.4%. This discrepancy in mortality rates across secondary intervention strategies highlights the need for more comprehensive investigations to refine bleeding management and improve survival in patients experiencing recurrent bleeding [3].

In our analysis, multivariate modeling identified several factors associated with poor prognosis, defined as rebleeding within 30 days or mortality. These included increases in both Rockall’s score and GB’s score (per 1‐point increase) and the presence of active bleeding at presentation. Univariate analysis indicated that also the increase in age by 10 years, history of cancer within 5 years, renal failure, and elevated heart ratio (per 10‐unit increase) were associated with worse outcomes; however, these factors did not remain significant in multivariate analysis. Similarly, Kim et al. [40] identified advanced age as well as RBC transfusion as key clinical predictors of mortality, while a Korean observational study found that age ≥ 65 years was an independent predictor of mortality [41]. The poorer outcomes observed in elderly patients may be due to a higher prevalence of comorbidities and increased susceptibility to physiological stressors. In turn, Güven et al. [38] reported that hemodynamic instability on admission (OR: 3.05, *p* = 0.006) and a history of malignancy (OR: 2.42, *p* = 0.029) were significant predictors of adverse outcomes. Moreover, they observed that the urgent endoscopy group required more endoscopic interventions (*p* = 0.006), whereas the early endoscopy cohort encountered increased transfusion needs and longer hospital stays (*p* = 0.002 and *p* = 0.040, respectively), findings that contrast with our observations [38]. These discrepancies could stem from the fact that patients in our urgent and early groups were more homogeneous in terms of the severity of general symptoms and pathologies within the stomach and duodenum. Another retrospective observational study found significant associations between presence of malignancy (OR: 3.58, 95% CI: 1.33–9.62), cirrhosis (OR: 4.67, 95% CI: 1.85–11.76), urgent endoscopy (OR: 0.36, 95% CI: 0.14–0.95), failed primary endoscopic treatment (OR: 15.03, 95% CI: 4.63–48.82), rebleeding (OR: 2.77, 95% CI: 1.03–7.45), and higher mortality [3]. These findings collectively highlight the complex interplay between patient characteristics, comorbidities, and procedural factors in determining outcomes after UGIB. When considering early endoscopy for patients with UGIB, it becomes imperative to address any hemodynamic instability [10]. Furthermore, optimizing pertinent comorbidities, correcting coagulopathy, and stabilizing hemodynamics before the endoscopic procedure are crucial to ensuring safety and efficacy. A prospective cohort study demonstrated that among hemodynamically stable patients (with the American Society of Anesthesiologists physical status ranging from 3 to 5), endoscopy performed between 12 and 36 h after presentation was associated with diminished in‐hospital mortality (OR = 0.48, 95% CI: 0.34–0.67) [42]. Conversely, in hemodynamically unstable patients, the optimal window for endoscopic intervention appeared to be 6 to 24 h postpresentation (OR = 0.73, 95% CI: 0.54–0.98). This “U‐shaped” relationship in these cohorts suggests that the first hours of hospitalization should be utilized for patient resuscitation and correction of reversible conditions, such as severe anemia, coagulopathy, or infections, prior to endoscopy. Nevertheless, the authors emphasize that endoscopy should remain a priority for patients with persistent severe hemodynamic instability refractory to intensive resuscitative efforts [42].

International consensus group recommends performing endoscopy within 24 h of presentation in patients with acute UGIB [5]. However, these guidelines do not provide a definitive stance regarding the potential benefit of performing endoscopy within 12 h in patients considered to be at high risk. Numerous observational studies, randomized controlled trials, and systematic reviews have shown that urgent endoscopy (variably defined as within 2 h to 12 h after presentation) does not significantly reduce mortality in unselected patients with acute UGIB [29, 43–47]. However, evidence from selected cohorts suggests that performing endoscopy within 12 h may confer better outcomes in patients with a higher risk of ongoing or recurrent bleeding [3]. In our study, after propensity‐score matching to balance the full set of baseline characteristics, none of the individual predictors initially associated with urgent endoscopy, such as active bleeding, abnormal vital signs, alcohol abuse, risk scores, comorbidities, or medication use, remained independently significant. Before matching, some of these factors appeared to influence clinicians’ decisions, but once patients were matched across their overall risk, it became apparent that the composite clinical context, rather than any single variable, primarily determined the timing of endoscopic intervention. This highlights the complex and multifactorial nature of decision‐making in real‐world management of UGIB. Interpreting results across studies remains challenging, given substantial heterogeneity in study design, follow‐up duration, patient selection, and types of endoscopic therapy employed.

### 4.1. Limitations

This study has several limitations. First, its retrospective design inherently restricts the level of evidence. However, it must be pointed out that conducting a prospective randomized trial in this context would pose ethical challenges, as delaying endoscopy in unstable or hypotensive patients for randomization purposes could jeopardize patient safety. Second, our institution offers a 24‐h, 7‐day endoscopy service, which may not be available in all healthcare settings. Consequently, our findings may not be directly generalizable to centers without continuous endoscopic coverage. Moreover, there were significant baseline imbalances between treatment groups (age, gender, HR, hypertension, and ulcer history), which may introduce residual confounding despite adjustment. A further limitation lies in the substantial proportion of patients (approximately 40%–50%) in whom no definitive bleeding source was identified during endoscopy, categorized as “other.” This heterogeneous group likely included cases of spontaneously arrested bleeding, lesions beyond the reach of standard upper endoscopy, or subtle mucosal abnormalities not visible during examination. Such heterogeneity may have diluted associations between specific bleeding etiologies and clinical outcomes, thereby reducing statistical power to detect significant differences between groups. Despite these limitations, a key strength of our analysis is the inclusion of all patients at high predicted risk for rebleeding or death, including those with persistent hypotensive shock despite initial resuscitation. This comprehensive inclusion enhances the clinical relevance of our findings and supports their applicability to the broader population of patients requiring urgent endoscopic evaluation for ongoing gastrointestinal hemorrhage.

**Nomenclature**
APC: Argon plasma coagulation
BP: Blood pressure
Cl: Confidence interval
ED: Emergency departments
GB’s score: Glasgow–Blatchford’s score
Hgb: Hemoglobin
INR: International normalized ratio
NSAIDs: Nonsteroidal anti‐inflammatory drugs
OR: Odds ratio
pRBCs: Packed red blood cells
SD: Standard deviation
UGI: Upper gastrointestinal
UGIB: Upper gastrointestinal bleeding

## Disclosure

All authors read and approved the final manuscript.

## Conflicts of Interest

The authors declare no conflicts of interest.

## Author Contributions

All authors contributed to the study conception and design. Material preparation, data collection and analysis were performed by Katarzyna Stasik, Katarzyna Ferenc, Krystian Partyka,, Wojciech Kilisiński, and Rafał Filip. The first draft of the manuscript was written by Rafał Filip and Katarzyna Stasik, and all authors commented on previous versions of the manuscript.

## Funding

The study received no funding.

## Supporting Information

Supporting Table 1. Baseline covariate balance: standardized differences before and after propensity‐score matching.

Supporting Table 2. Adjusted odds ratios (95% Cl) from multivariable logistic regression in the propensity‐score–matched sample.

Figure S1. Adjusted odds ratios (95% CI) for urgent endoscopy in the propensity‐score–matched cohort.

## Supporting information


**Supporting Information** Additional supporting information can be found online in the Supporting Information section.

## Data Availability

The data that support the findings of this study are available from the corresponding author upon reasonable request.
