# Supplementary material for: Impact of Urgent Versus Early Endoscopy on Outcomes in Acute Upper Gastrointestinal Bleeding: A Retrospective Study
Source: Can J Gastroenterol Hepatol. 2025 Nov 30;2025:4328051. doi: 10.1155/cjgh/4328051 (PMC12665192; doi:10.1155/cjgh/4328051)
Supplement: Supplementary file 1 — Supporting Information Additional supporting information can be found online in the Supporting Information section. [file CJGH-2025-4328051-s001.docx]

**Supplementary material**

We performed a propensity‐score–adjusted logistic regression (stratifying by PS quintiles) to account for the observed imbalance. Active bleeding: OR 1.39 (95% CI 0.74–2.58), *p* = 0.30; heart rate (per 10 bpm): OR 1.03 (95% CI 0.88–1.21), *p* = 0.72 and alcohol abuse: OR 1.67 (95% CI 0.85–3.30), *p* = 0.14 were adjusted for baseline risk via the propensity-score quintiles. Overall, once we balance on the joint distribution of age, sex, vitals, comorbidities, and scores, none of the previously significant univariate predictors remain statistically significant, highlighting the importance of accounting for confounding.

**Supplementary table 1. Baseline covariate balance: standardized differences before and after propensity-score matching**

| **Variable** | **Standard difference before** | **Standard difference after** |
| --- | --- | --- |
| **Age (10 years)** | -0.171 | 0.021 |
| **Heart rate (each 10 beats-per-minute increase)** | 0.267 | -0.083 |
| **Active bleeding** | 0.345 | -0.108 |
| **Alcohol abuse** | 0.318 | 0.183 |
| **Rockall’s score (points)** | -0.07 | 0.109 |
| **Glasgow-Blatchford score (points)** | 0.135 | 0.057 |
| **Renal failure** | -0.032 | 0.081 |
| **Tumor within 5 years** | 0.015 | 0.081 |
| **History of stomach ulcers** | 0.074 | -0.119 |
| **NSAIDs** | -0.169 | -0.265 |
| **Aspirin** | -0.067 | 0.0 |
| **Warfarin** | -0.098 | 0.0 |
| **Hgb at admission** | -0.015 | -0.0038 |

**Supplementary table 2.** **Adjusted odds ratios (95% Cl) from multivariable logistic regression in the propensity-score–matched sample**

| **Variable** | **OR** | **2.5% CI** | **97.5% CI** | **p** |
| --- | --- | --- | --- | --- |
| **Const.** | 2.596 | 0.109 | 61.79 | 0.555 |
| **Age (10 years)** | 0.934 | 0.736 | 1.185 | 0.574 |
| **Heart rate (each 10 beats-per-minute increase)** | 0.871 | 0.704 | 1.079 | 0.206 |
| **Active bleeding** | 0.702 | 0.345 | 1.429 | 0.329 |
| **Alcohol abuse** | 1.598 | 0.725 | 3.523 | 0.245 |
| **Rockall’s score (points)** | 1.076 | 0.881 | 1.316 | 0.471 |
| **Glasgow-Blatchford score (points)** | 1.039 | 0.912 | 1.183 | 0.569 |
| **Renal failure** | 1.325 | 0.397 | 4.426 | 0.647 |
| **Tumor within 5 years** | 1.748 | 0.150 | 20.367 | 0.655 |
| **History of stomach ulcers** | 0.600 | 0.191 | 1.881 | 0.381 |
| **NSAIDs** | 0.376 | 0.093 | 1.515 | 0.169 |
| **Aspirin** | 0.806 | 0.097 | 6.696 | 0.841 |
| **Warfarin** | 0.944 | 0.173 | 5.127 | 0.947 |
| **Hgb at admission** | 1.024 | 0.881 | 1.189 | 0.761 |

**Figure S1. Adjusted odds ratios (95% CI) for urgent endoscopy in the propensity-score-matched cohort.
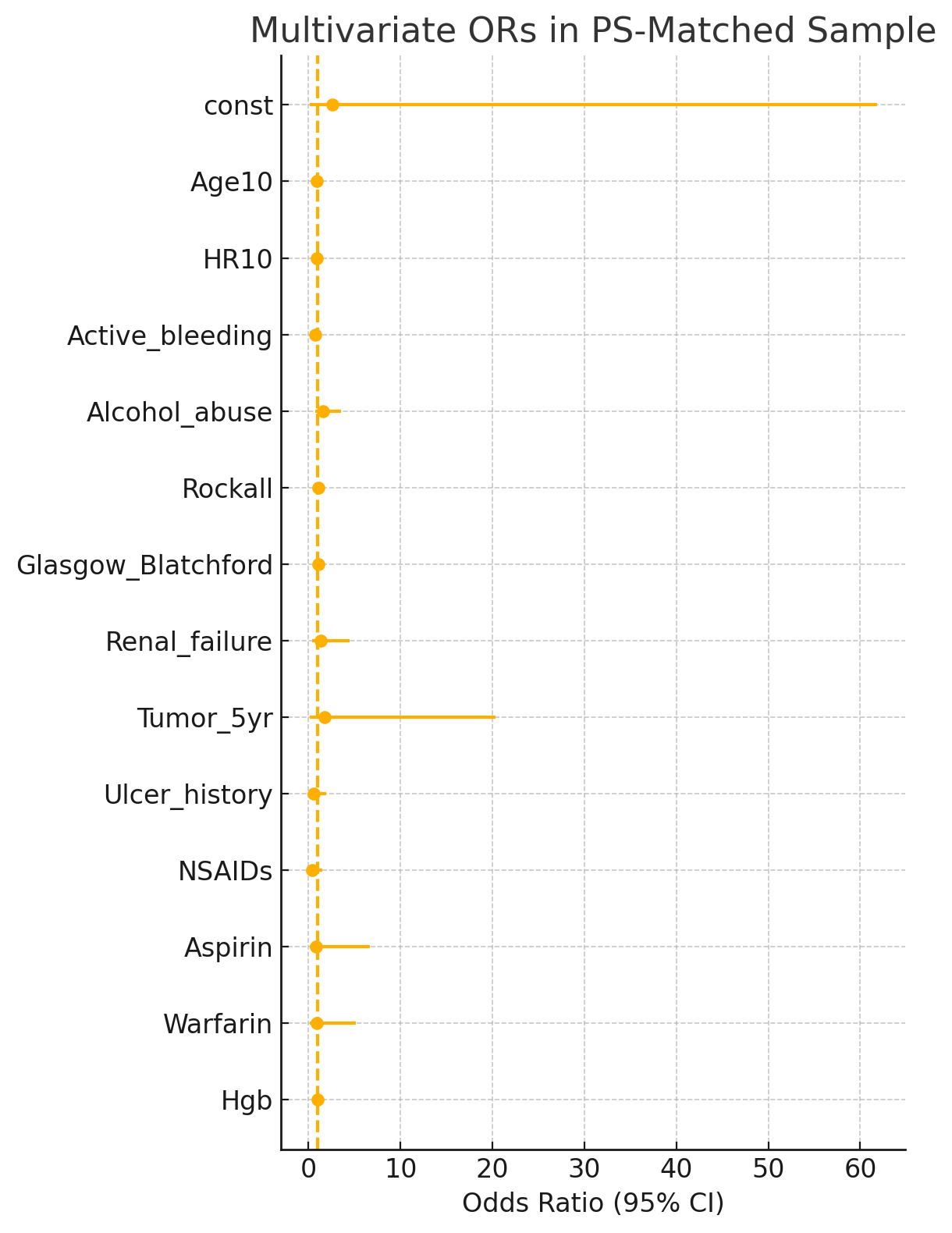
**

Once we balance on the full set of baseline covariates via propensity‐score matching (and even when varying the caliper), none of the parameters we tested (active bleeding, heart rate, alcohol abuse, scores, comorbidities, medications, etc.) reach statistical significance in predicting an urgent endoscopy decision.

Before matching, certain factors appeared to drive clinicians toward an urgent scope. But after we compare like-with-like patients (those with very similar overall risk profiles), those individual factors no longer stand out. This suggests that it was not any single variable in isolation but the overall patient risk constellation that was guiding timing decisions.
